# Depletion of Regulatory T Cells Induces High Numbers of Dendritic Cells and Unmasks a Subset of Anti-Tumour CD8^+^CD11c^+^ PD-1^lo^ Effector T Cells

**DOI:** 10.1371/journal.pone.0157822

**Published:** 2016-06-24

**Authors:** Nicolas Goudin, Pascal Chappert, Jérome Mégret, David-Alexandre Gross, Benedita Rocha, Orly Azogui

**Affiliations:** 1 Plateau technique de Cytometrie et d’Imagerie Cellulaire, Structure Fédérative de Recherche Necker, INSERM US 24-CNRS, UMS 3633, Paris, France; 2 Institut Necker Enfants Malades, INSERM U1151, CNRS, UMR8253, Faculté de Médecine, Université Paris Descartes, Sorbonne Paris Cité, Paris, France; Weizmann Institute of Science, ISRAEL

## Abstract

Natural regulatory T (Treg) cells interfere with multiple functions, which are crucial for the development of strong anti-tumour responses. In a model of 4T1 mammary carcinoma, depletion of CD25^+^Tregs results in tumour regression in Balb/c mice, but the mechanisms underlying this process are not fully understood. Here, we show that partial Treg depletion leads to the generation of a particular effector CD8 T cell subset expressing CD11c and low level of PD-1 in tumour draining lymph nodes. These cells have the capacity to migrate into the tumour, to kill DCs, and to locally regulate the anti-tumour response. These events are concordant with a substantial increase in CD11b^+^ resident dendritic cells (DCs) subsets in draining lymph nodes followed by CD8^+^ DCs. These results indicate that Treg depletion leads to tumour regression by unmasking an increase of DC subsets as a part of a program that optimizes the microenvironment by orchestrating the activation, amplification, and migration of high numbers of fully differentiated CD8^+^CD11c^+^PD1^lo^ effector T cells to the tumour sites. They also indicate that a critical pattern of DC subsets correlates with the evolution of the anti-tumour response and provide a template for Treg depletion and DC-based therapy.

## Introduction

Accumulating evidence, in both humans and mice, indicates that specific immune responses to tumours require the activation, amplification, and cytotoxic function of antigen-specific T cells. Notably, a strong infiltration of CD8 T cells at the tumour site is needed to control tumour growth [1]. However, tumour-specific responses are usually not sufficient to eradicate tumours. This inadequate anti-tumour response is due to several mechanisms of peripheral tolerance that control different stages of the immune response leading to incomplete differentiation of anti-tumour CTLs [2]. These tolerogenic mechanisms include regulatory T cell-mediated suppression [3], and insufficient activation or functional inactivation of tumour-specific lymphocytes by overexpression of CTLA-4 or PD1 negative receptors [4–6]. All these events lead to low effector T cell numbers, inadequate tumour infiltration, and subsequent tumour growth.

Suppression of immune responses by thymus-derived CD4^+^CD25^+^Foxp3^+^Tregs (Tregs) is a well-documented mechanism of tolerance [7, 8]. Foxp3 is an essential transcription factor for the development and function of Tregs [9]. Mechanisms of Treg-mediated suppression include the production of IL-10, TGF-ß [10, 11], and the expression of anti-co-stimulatory molecules such as CTLA-4. More recently, a regulation loop between Tregs and dendritic cells (DCs) was demonstrated [12], where Treg ablation in Foxp3^*DTR*^ mice was shown to induce the differentiation of high numbers of pre-DCs and DCs, and their accumulation in LNs [13, 14]. Lastly, it was shown that Tregs suppressed immune responses by preferentially forming aggregates with DCs limiting their expression of co-stimulatory receptors CD80 and CD86 [15] and the availability of IL-2 in the microenvironment [16], both required for the generation of effector T cells. However, none of these experiments were performed in tumour-bearing mice. Thus, insights concerning the dominant *in vivo* mechanism involved in the Treg-mediated suppression of anti-tumour responses is still lacking and could be pivotal for the specific manipulation of Tregs.

The role of Tregs in the suppression of the anti-tumour response was first demonstrated when the administration of a single dose of anti-CD25 antibodies (PC61) prior to tumour injection, induced tumour regression in the majority of treated mice [17]. In another model of tumour-bearing mice, we previously showed that elimination of CD25^+^Treg resulted in the strong activation/amplification of CD4 and CD8 effector T cells and the control of tumour growth [18]. However, in spite of a plethora of reports describing how Tregs exert their function on conventional T cells, it is unclear how this suppression impacts the immune response in tumour-bearing mice, and how Treg depletion promotes tumour infiltration by T cells, mediating its destruction. Most studies of the effects of Tregs depletion on tumour rejection focused the immune response in the draining lymph node (DLN) or at the tumour site, but a correlation between these two necessary events is not well documented. In vivo imaging of cytotoxic antigen-specific TCR-Tg cells (Tg-CTL) infiltrating a solid tumour expressing the cognate antigen showed that tumour regression requires CTL motility and profound tumour infiltration, and is dependent on the presence of antigen [19]. However, in non-transgenic mice, the antigens expressed by tumours are more diverse, and the predominant populations available to control tumour growth are believed to be low avidity T cells. Identification of cell surface markers or other characteristics expressed by tumour-infiltrating CD8 T cells in a normal T cell repertoire would represent a more selective target to identify particular T cell subsets that can more efficiently promote tumour infiltration and regression.

We used here the 4T1 mammary carcinoma and PC61-mediated Treg depletion as an experimental approach that allowed us to study the mechanism of tumour regression, with anti-tumour responses being evaluated simultaneously in the DLN and at the tumour site. We show that Treg depletion induced the sequential recruitment and expansion of the two main DC subsets, and gave rise to the expansion of cytotoxic CD8^+^ T cells in DLNs, characterized by their expression of CD11c and low levels of PD1. Tumour neo-angiogenesis was fully modified, allowing the direct access of these CD8^+^CD11c^+^ T cells from the DLNs to the tumour sites and elimination of the tumour in treated mice. When low numbers of these cells were injected i.v. in non-treated tumour bearing mice, they were able to control tumour growth. Additionally, tumour neo-angiogenesis appeared enhanced favoring direct access of CD8^+^CD11c^+^ effector T cells from the DLNs to tumour sites and elimination of the tumour in treated mice. Thus, our data demonstrates an unappreciated effect of Treg depletion in a coordinated expansion of DC subsets in tumour bearing mice, and suggest that the efficacy of therapeutic vaccines requires the partial depletion of Tregs allowing to increased number of DCs and the generation of large numbers of CD8^+^ CD11c^+^ PD1^lo^ T cells, which have the capacity to infiltrate the tumour and control its growth.

## Materials and Methods

### Mice, 4T1 tumour cells and injections

All animal experiments were conducted according to European Directive (2010/63/UE) and were approved by the Ethical Committee of Paris Descartes University (registered number: 14–075). 6 to 8 week-old BALB/c mice were purchased from Charles River Laboratories. The 4T1 tumour cell line derived from a BALB/c spontaneous mammary carcinoma was supplied by Dr S. Ostrand-Rosenberg (Baltimore University). 4T1 cells were cultured in RPMI supplemented 5% FCS and 1% antibiotics. 2x10^5^ cells were injected s.c. in the left flank of the mice in a final volume of 100μl PBS. Tumours size was measured by caliper every 3 days. After 4, 7, 10 and 14 days post-injection, mice were sacrificed and tumours resected. To isolate lymphocytes from tumour tissues, tumours were collected, cut into small pieces, and incubated in RPMI with 0.15 mg/mL Liberase TL (Roche) and 200ng DNase (Roche) for 45 min at 37°C. Tumour tissues were then homogenized until resolved into a cell suspension.

For Treg depletion, mice were treated i.p. with 300μg anti-CD25 antibody PC61 (BioXcell, West Lebanon, USA) 4 days before tumour inoculation.

### Identification of dendritic subsets and T cells

For flow cytometry analysis, the inguinal lymph node from the tumour (left) was taken as the tumour-draining lymph node (DLN) and the contralateral inguinal lymph node as a non-draining lymph node (nDLN). LNs and tumours were excised at various time-points, cut into pieces, and digested into single cell suspensions using collagenase D1 (1mg/ml) and DNAse (100ng/ml)(Roche Applied Science) for 15–40 minutes at 37°C. T cells. DC subsets were then treated with EDTA 5mM for 5 min and stained with anti-CD11c (PE-Cy7), anti-MHC-II (FITC), anti-CD8α (Pacific Blue), anti-CD11b (PerCP-Cya5.5), anti-DEC-205 (PE) and anti-CD103 (APC) antibodies. To further delineate DC subsets, total cells were stained with anti-CD3 (APC), CD19 (APC), and B220 (APC). T cell subset was stained with anti-CD8β-PE or CD8β-FITC.

### Intracellular staining for Foxp3 and KI67 expression

Treg characterization and KI67 proliferation were assessed using a Foxp3 permeabilisation kit (eBioscience). Cell surface staining was performed with anti-CD4 (APC-AlexaFluor-780), anti-CD3 (PerCP-Cya5.5) and anti-CD25 (clone 7D4-FITC). Intracellular staining was performed with anti-Foxp3 (PE) or anti-KI67 (PE).

### In vitro proliferation assay

CD11c^hi^MHCII^int^CD11b^hi^ sorted by using a cell sorter FACS Aria I (Becton Dickinson) from DLNs and tumours from untreated and treated mice. DCs (3x10^3^) and CellTrace violet (CTV) labeled CD8 T cells (3x10^4^) sorted from normal mice LNs were cultured for 3 days in 96 well round-bottomed plates (Costar) in RPMI 1640 medium supplemented with 5% FCS, penicillin (100U/ml), streptomycin (100μg/ml) and 50 μM 2-ME. Sorted CD4^+^CD25^-^ T cells were added to the culture separately. Anti-CD3 mAb (145-2C11) at a final concentration of 0.1 μg/ml was added to the cultures for stimulation. CTV dilutions were used to determine the degree of CD8 T cell proliferation. After 3 days, cells were collected, treated with 5 mM EDTA and stained with anti-PD-1 mAb.

### In vitro degranulation assay

CTL granule exocytosis was measured as mobilization of the lysosomal marker CD107a (Lamp-1) to the cell surface as described [20]. To trigger degranulation of CD107a on the cell surface, sorted CD8^+^CD11c^+^ tumour-draining LNs were harvested on day 14 and incubated with sorted CD11c^hi^ dendritic cells, PE-conjugated CD107a mAb, and monensin for 6 hours at 37°C. Cell suspensions were then stained for CD3 and CD8β to identify CTLs, as well as CD107a and analysed by flow cytometry.

### Single-cell sorting and RT-PCRs for cytotoxic-associated gene amplification

CD3^+^CD8^+^CD11c^+^ single T cells were directly sorted into PCR tubes after staining with anti-CD3 FITC, anti-CD8β Pacific Blue and anti-CD11c Percp-Cy5-5 Abs. cDNA synthesis and PCR amplification with specific primers of CD3, CD11c, *IFN*γ, *Granzyme B* (*Grzb*) and *Perforin* (*Prf*) genes (Table 1) were performed according to the method already described [18].

**Table 1:** Specific primers used for single-cell RT-PCR.

Primers	5' Forward	3' anti-sens	3' nested
CD3	GCCTCAGAAGCATGATAAGC	CCTTGGCCTTCCTATTCTTG	TGACCATCAGCAAGCCCAGA
CD11c	GCTGTACCTGGATAGCCTTTCTTC	GTTCTGGCTCTGCTTGAATG	CAGGGATATGTTCACAGCCTCT
IFNγ	ACACTGCATCTTGGCTTTGCAGCT	CGACTCCTTTTCCGCTTCCT	CACTCGGATGAGCTCATTGAATGC
GrzB	GATGAAGATCCTCCTGCTACTGCT	CCAACCAGCCACATAGCACACAT	CACATCTCCTGGCTTCACATTGAC
Prf	CAGCTGAGAAGACCTATCAGGACC	TACTTCGACGTGACGCTCACGGTA	GTATTCACTGGAGACGCTGGCTTG

### Histology and confocal laser microscopy

Inguinal draining LNs and tumours were harvested at day 12 after tumour inoculation, directly frozen in OCT and stored at -80°C. Multiple LN and 6 μm tumour sections were then prepared with a cryostat. Sections were fixed with 50% Acetone and 50% Ethanol (10 min at -20°C) and washed with PBS. Sections were blocked over night with Blocking Buffer Low Protein (eBioscience) and then stained with appropriate primary and secondary antibodies. Each incubation step was conducted for 2 hours in the dark at room temperature. The sections were first stained with monoclonal antibodies: purified anti-CD8β and biotinylated-CD11c (clone HL3), secondly stained with anti-rat Alexa fluor 555, Cy5-conjugated streptavidin, and Alexa fluor 488- CD31. Antibodies were purchased from Biolegend, BD Pharmingen, and eBioscience. Images were acquired using a Zeiss LSM 700 confocal microscope with ZEN 2009. Sections were analysed by ImageJ v1.47 software. Levels of angiogenesis were determined by quantitating the number of vessel on the tumour surface and the percentage of CD31-positive pixels per area of tumour.

### Statistical analysis

Means of two groups were compared with two-tailed Student t test for unpaired observation with Prism 4 software (Graphpad software, inc) and also by Fisher’s exact test using C-Stat 1.0 (Oxtech, Oxford, UK). Differences with p values < 0.05 were considered significant.

## Results

### Transient Treg depletion is sufficient to induce tumour regression

**Fig 1:**
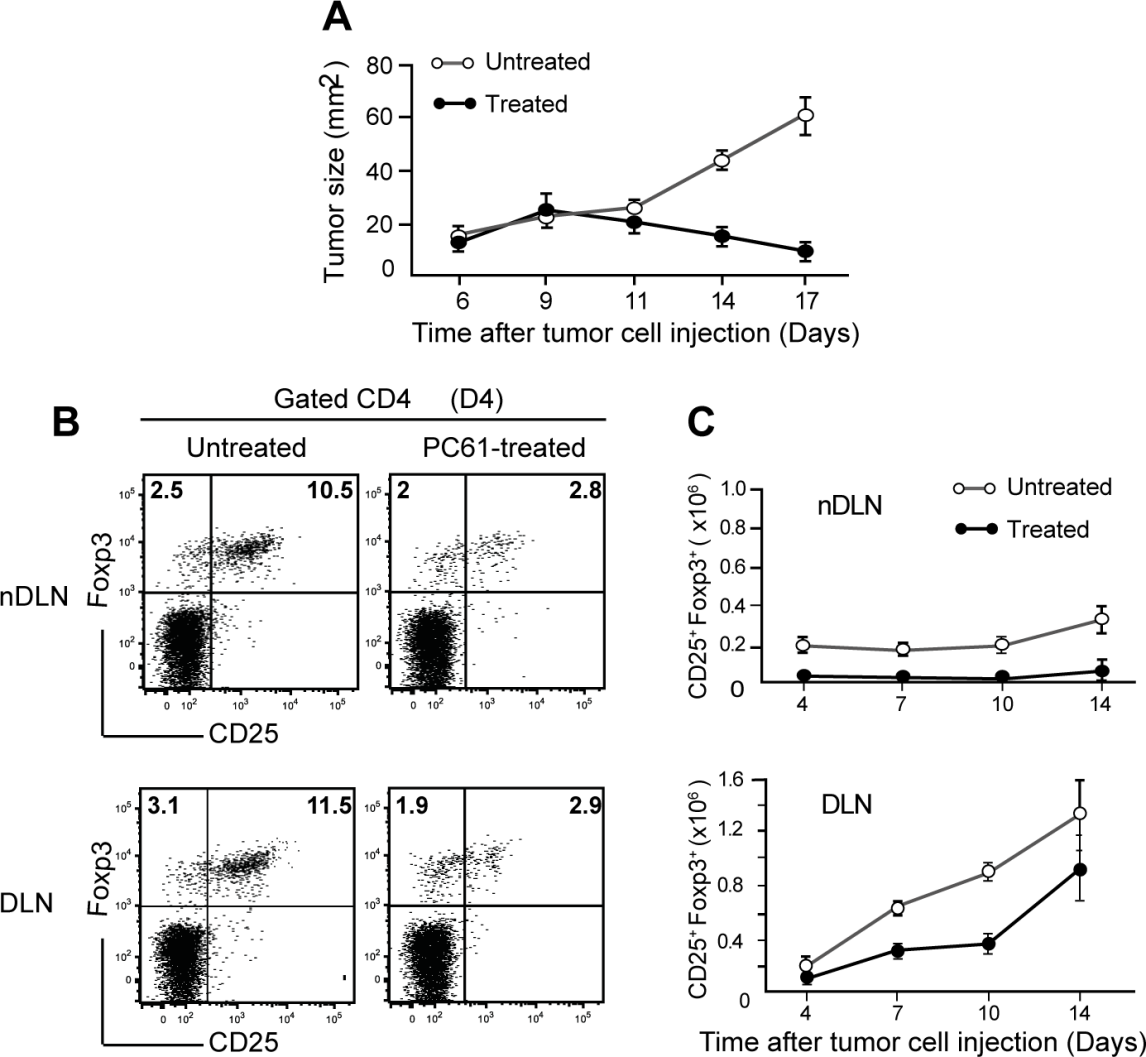
Partial depletion of CD25^+^Foxp3^+^ Tregs induces complete tumour regression. Balb/c mice were injected i.p. with 300μg of PC61 antibodies and 2x10^5^ 4T1 tumour cells were inoculated subcutaneously 4 days later. (A) Graph shows growth of 4T1 tumours in untreated (n = 10) and PC61 treated (n = 20) mice. (B) Representative plots of CD25 and Foxp3 expression in CD4 T cells from tumour non-draining LN (nDLN) and Draining LN (DLN) 4 days after tumour cell injection. (C) CD25^+^Foxp3^+^ Treg cell numbers were evaluated in nDLN and in DLN at the indicated time points after tumour injection. Bars show mean values ± SEM. Data are representative of more than three independent experiments.

We previously reported that the prophylactic depletion of Tregs using anti-CD25-specific antibodies (PC61) resulted in a partial depletion of Tregs and the complete regression of the 4T1 mammary carcinoma in majority of tumour-bearing mice [18]. The 4T1 murine carcinoma cell line is known to express significant levels of MHC class I molecules spontaneously [21]. This tumour is an animal model for stage IV human breast cancer as tumour growth and metastatic cells spread very closely mimic human breast cancer. This tumour cell line induces metastasis even after injection of low numbers as 10^5^ to 2 10^5^ cells per mouse. To gain quantitative information on tumour growth and the different cellular subsets involved in tumour regression, we first depleted CD25^+^ Tregs by one injection of PC61 antibodies in a group of 20 mice. 4 days later, PC61-treated and 10 untreated mice were injected subcutaneously in one flank with 2 x 10^5^ 4T1 mammary carcinoma cells. Tumour sizes were measured every 3 days until complete tumour regression. Comparison of tumour sizes between treated and untreated mice showed similar kinetics of tumour growth until days 9–11 after injection of tumour cells (Fig 1A). Thereafter, tumour size increased in non-treated mice, while tumours started to regress and completely disappeared between days 20–25 in 80% (16/20) of treated mice. To determine whether failure to cure all mice was due to inefficient depletion of CD25^+^ Tregs, the level of CD25^+^ Treg depletion was evaluated in the blood of treated mice before tumour inoculation. We found the same level of Treg reduction in the blood of mice bearing regressive and non-regressive tumours (data not shown). These data indicate that all treated mice had the same level of Treg depletion before tumour inoculation.

To investigate the rate of Tregs repopulation following PC61 treatment in tumour- bearing mice, we compared cell numbers in non-draining LNs (nDLN) and in the draining LNs (DLN) at different time points after tumour inoculation. In nDLNs, the percentages and absolute numbers of CD25^+^Foxp3^+^ Tregs were reduced by 76% in all treated mice, and this depletion lasted a minimum of 14 days (Fig 1B). Conversely, we detected a robust anti-tumour Treg response in the DLNs of both untreated and treated mice with equivalent numbers by day 14 (Fig 1C). However, up to 10 days after injection of tumour cells the number of Tregs in the DLN of treated mice was still 2-fold reduced as compared with untreated mice. Thus, the kinetic analysis of Treg response in DLN of mice that were treated with PC61 revealed the transient effect of the depletion, despite the sustained tumour regression observed. The late accumulation of high numbers of CD25^+^Foxp3^+^ Tregs cells (D14) in the DLN of treated mice indicates that Tregs suppressive effects are likely to occur mainly at the initial phase of the anti-tumour response.

### Treg depletion increases the number of CD11b^+^ resident dendritic cell subsets in the tumour draining-LNs

**Fig 2:**
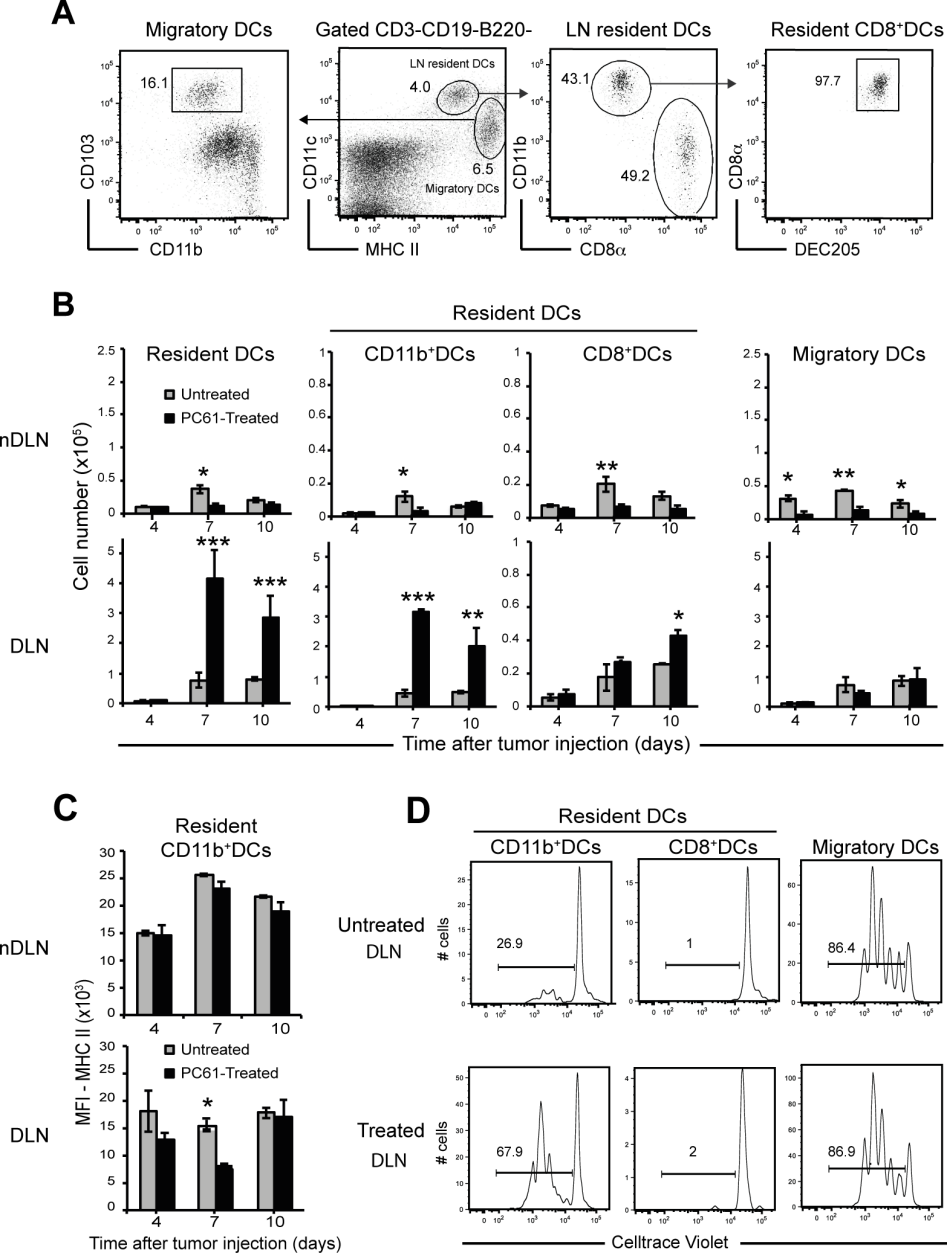
Treg depletion induces high numbers of CD11b^+^ DCs in tumour-draining LNs. Groups (n = 3) of untreated and PC61-treated mice were injected s.c. with 2x10^5^ 4T1 tumour cells and DC subsets later analyses. (A) Gating strategy for DC subsets in normal LNs. Lineage^-^ cells (CD3ε^-^ CD19^-^B220^-^) were examined for CD11c and MHC-II expression to quantify lymphoid-resident DCs (CD11c^hi^MHC-II^int^) and migratory DCs (CD11c^int^MHC-II^hi^) (center panel). Resident DCs were further gated for the expression of CD11b^hi^ and CD8^+^ and DEC205^+^ (right panel). Migratory DCs were further gated into CD103^+^ DCs (left panel). (B) Numbers of resident DCs, migratory DCs and CD11b^hi^, CD8^+^ subsets belonging to the resident DCs in tumour non-draining LNs (nDLN) and draining LNs (DLN) at indicated time after injection of tumour cells. (C) Comparison of the Mean Fluorescence Intensity (MFI) of the MHC II on CD11b^+^ subsets studied at day 7 in the indicated organs. (D) Histograms show the proliferation of CellTrace violet-labeled naïve CD8 T cells stimulated by DC subsets sorted at day 7 in the indicated organs with the gates shown in (A). Data are representative of two independent experiments for the kinetics and for times at day 7. Bars show mean values ± SEM. *,p<0.05, **,p<0.001, ***,p<0.0001. P values were calculated using Student’s t test.

Dendritic cells (DCs) are promising targets for immunotherapy because they play an essential role in initiating and regulating T cell immunity. It has been shown that CD25^+^Foxp3^+^ Tregs led to increased numbers of LN-resident DCs [14] but neither the impact of the Treg depletion on the different DC subsets nor the cell-mediated response to tumour cells have not been examined in details. To address this issue in the context of tumour regression versus tumour progression, PC61-treated and untreated mice were injected with 4T1 cells and DC numbers were evaluated by performing a kinetic analysis of each subset in tumour draining LNs (DLNs) and non-draining LNs (nDLNs). The gating strategy to identify these subsets was already described [22, 23] and is shown in Fig 2A. Live lineage^-^ cells were examined for CD11c and MHC II expression characteristic of the main subsets of CD11c^+^ DCs, i.e, CD11c^hi^MHC II^int^ LN resident DCs including CD11b^hi^, CD8^+^DEC205^+^ and CD11c^hi^MHC II^hi^ including CD103^+^ migratory DCs (mDCs).

We found that PC61 treatment led to reduced numbers of the three DC subsets at all time points studied in nDLNs (Fig 2B). In contrast, the number of resident DCs appeared strikingly increased in the DLN of treated mice albeit with a different kinetics of accumulation for each DC subset. We observed a 7 and 4-fold increase of resident CD11b^hi^ DCs at day 7 and day 10 after tumour injection while increased numbers of CD8^+^ DCs were only observed at day 10 (Fig 2B). Since Treg depletion also associates with a major increase in the size of the DLNs (see below), we analysed CD11b^hi^ DCs expression of MHC II, which is well associated with the maturation status of DCs. Notably, as assessed by staining profiles and mean fluorescence intensity (MFI) of each staining. The level of MHC II expression on CD11b^hi^ DCs from treated mice was lower than the level of MHC II of DCs from untreated mice at day 7 (Fig 2C). To compare the ability of the distinct DC subsets to activate naïve CD8 T cells, we next compared CD8 T cell proliferation upon *in vitro* culture in the presence of DCs isolated from untreated and PC61-treated mice. 7 days after tumour cell injection, purified CD11b^hi^, CD8^+^ and mDCs were separately co-cultured with CellTrace violet labeled CD8 T cells and cell divisions analysed by Flow cytometry (Fig 2C). Since not all DCs present tumour antigens, anti-CD3 MoAbs were added to all cell cultures. Surprisingly, CD8^+^ DCs did not induce CD8 T cell proliferation while mDCs isolated from both groups stimulated cell proliferation at the same level. Conversely, CD11b^hi^ DCs were more efficient at stimulating the proliferation of CD8 T cells than those isolated from untreated mice (Fig 2D).

These data indicate that PC61 treatment followed by tumour cells injection induced an increase in LN-resident DC subsets in the DLNs. The greatest impact was observed in the CD11b^hi^ subset with a peak between day 7 and 10 while CD8^+^ DCs increased at day 10 only. Thus, each DC subset showed different kinetic trends of accumulation demonstrating that Tregs differentially regulate the number of DC subsets at different time points of the anti-tumour response. In addition, mDCs and CD11b^hi^ DCs were significantly more effective at stimulating the proliferation of CD8 T cells than were the CD8^+^ DCs.

### Treg depletion unmasks a subpopulation of cytotoxic effector CD8 T cells expressing CD11c in the DLN

**Fig 3:**
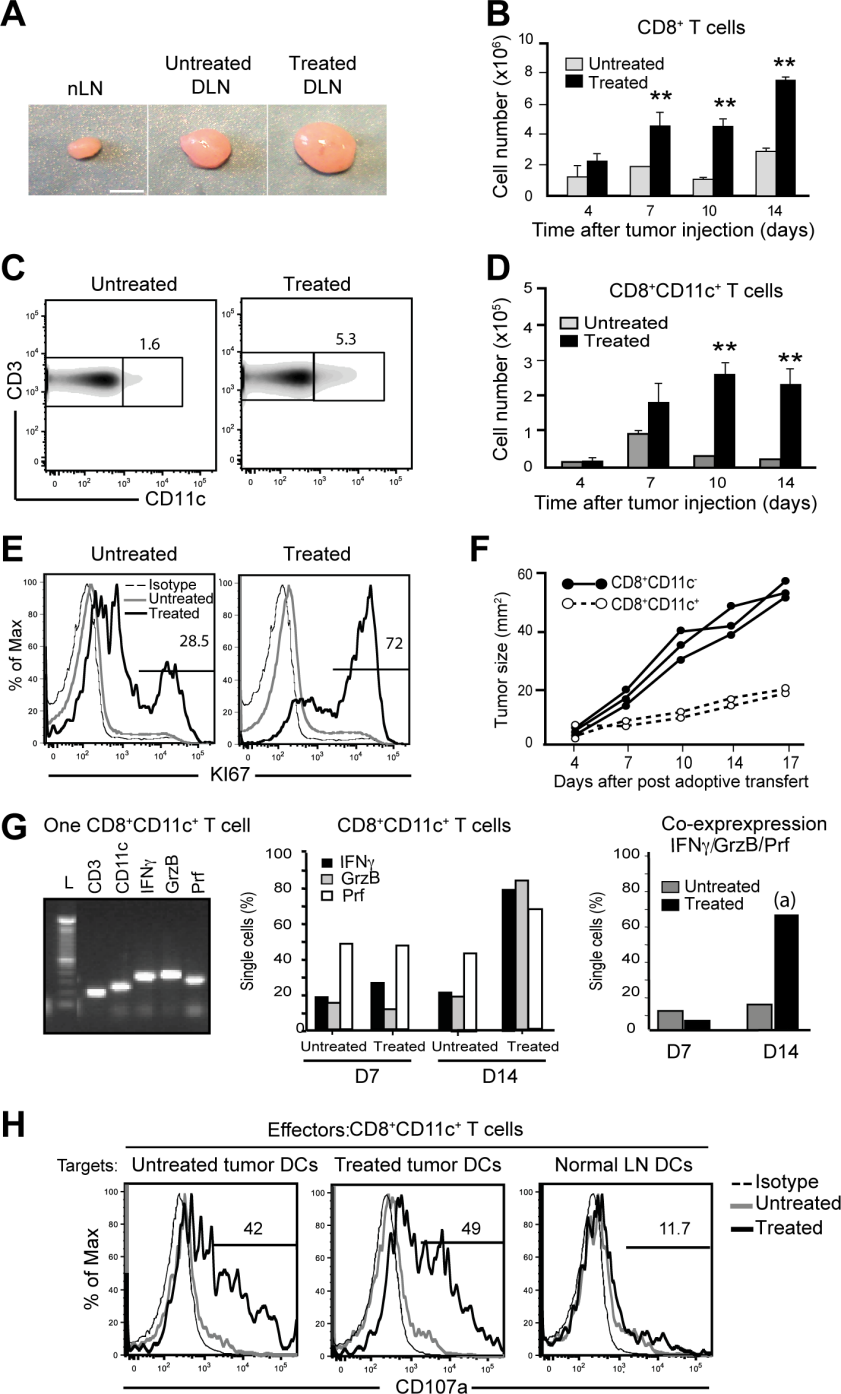
Treg depletion leads to increased cell numbers and functions of effector CD8 T cells expressing CD11c in tumour DLNs. (A) Photograph show the sizes of normal LN (nLN), untreated and PC61-treated DLNs. White bar represents 1mm. (B) Graph shows total cell numbers of CD8 T cells per LN in the two groups of mice (n = 3) at the indicated time after injection of tumour cells. (C) Flow cytometry data analysis of CD3^+^CD11c^+^ cells gated on total CD8 T cells of DLN from treated and untreated mice. (D) Graph shows total cell numbers of CD8^+^CD11c^+^ T cells in indicated groups studied at different time points after tumour cell injection. SEM is shown with error bars. Results are pooled from 5 experiments. (E) Flow cytometry determination of the frequency of CD8^+^CD11c^+^ and CD8^+^CD11c^-^ proliferating (KI67+) cells in the indicated groups (n = 3). A representative of two independent experiments is shown. (F) Tumour growth over time after CD8^+^CD11c^+^ T or CD8^+^CD11c^+^ T cell adoptive transfers into untreated mice. (G) Representative gel image showing RT-PCR products of indicated genes expressed in one CD8^+^CD11c^+^ T cell (left). Gene expression was analysed on a series of sorted CD3^+^CD8^+^CD11c^+^ single T cells from DLNs of untreated (120 cells) and PC61-treated mice (109 cells) (middle). Graph shows percentages of CD8^+^CD11c^+^ cells expressing each gene (middle). Graph shows percentages of CD8^+^CD11c^+^ cells co-expressing the four genes (right). (a) Differences between treated and untreated mice were analysed by Fisher’s exact test, p = 5.10^−3^ (H) Representative histogram of cell surface CD107a expression on CD8^+^CD11c^+^ T cells cultured with purified DCs obtained from untreated and treated tumours used as target cells. DCs purified from normal LN were used as control. **,p<0.001. P values were calculated using Student’s t test.

The impact of Treg depletion on anti-tumour CD8 T cell response in DLNs from both PC61-treated and untreated mice was studied at day 12 after tumour cell injection. First, degree of DLN enlargement was measured and showed different sizes in each group of mice (Fig 3A). While in untreated tumour-bearing mice the size of DLN was increased 2-fold compared with normal LN, we observed a 4-5-fold increase in the size of the DLN from Treg-depleted mice. The numbers of CD8 T cells were also increased, the most striking effects found between days 7–14 in the DLN of treated mice (Fig 3B).

When naïve CD8 T cells are primed and become effector cells, they undergo a shift in the expression of surface inflammatory receptors and newly expressed integrin such as the CD11c. Among the large and diverse CD8 T cells compartment in DLNs, the expression of the integrin CD11c changed during the course of tumour growth, and particularly during the phase of tumour regression. The percentages (Fig 3C) and absolute numbers of CD8^+^CD11c^+^ T cells (Fig 3D) increased in DLN of treated mice as compared with untreated mice. Further analysis of the CD8^+^CD11c^+^ population showed that it was significantly enriched in proliferating cells expressing KI67, a cell cycle-associated nuclear protein (Fig 3E).

To test their capacity to control tumour growth *in vivo*, CD8^+^CD11c^+^ T cells were further sorted from DLN of treated mice 14 days after tumour cell injection and 6x10^5^ cells were transferred to untreated mice, one day after 4T1 tumour inoculation. Analysis of tumour size every 3 days during 17 days showed that CD8^+^CD11c^+^ cells significantly reduced tumour growth (Fig 3F), indicating that these cells controlled tumour growth. However, the low number of transferred cells was not sufficient to completely eliminate the tumour cells.

Cytotoxic functions were also restricted to CD8^+^CD11c^+^ T cell subset. Indeed, RT-PCR analysis performed on a series of CD8^+^CD11c^+^ single cells showed that of the cytotoxicity-associated genes *IFN*γ, *Prf*, and *Grzb* were associated to CD3 and CD11c expression (Fig 3G, left). Analysis of co-expression of the four genes showed that CD8^+^CD11c^-^ T cells did not co-express these mRNAs (data not shown), while they were co-expressed by CD8^+^CD11c^+^ cells (Fig 3G, right). Moreover, while the frequency of cells co-expressing these mRNAs was low in DLN of both groups by day 7, the frequency of co-expressing T cells increased 6-fold in PC61 treated mice by 14 days after tumour inoculation. Additionally, only CD8^+^CD11c^+^ T cells were able to kill the sorted intra-tumour MHC-II^hi^CD11c^hi^ DCs (which should present tumour antigens), as determined by mobilization of the lysosome marker CD107a to the cell surface upon encounter with these targets (Fig 3H). These results show that expansion/differentiation of CD8^+^CD11c^+^ T cells peaks by day 14 after tumour inoculation, correlating with the kinetics of tumour elimination (Fig 1A).

Therefore, these results provide evidence that cytotoxic effector CD8 T cells in DLNs are highly enriched in the CD8^+^CD11c^+^ compartment. This effector compartment, which is much increased in PC61-treated mice contained cells that are actively dividing, co-express mRNAs coding for effector functions, and are able to kill the intra-tumour DC targets.

### Tumour infiltrates contained high frequency of CD8^+^CD11c^+^ T cells after Treg depletion

**Fig 4:**
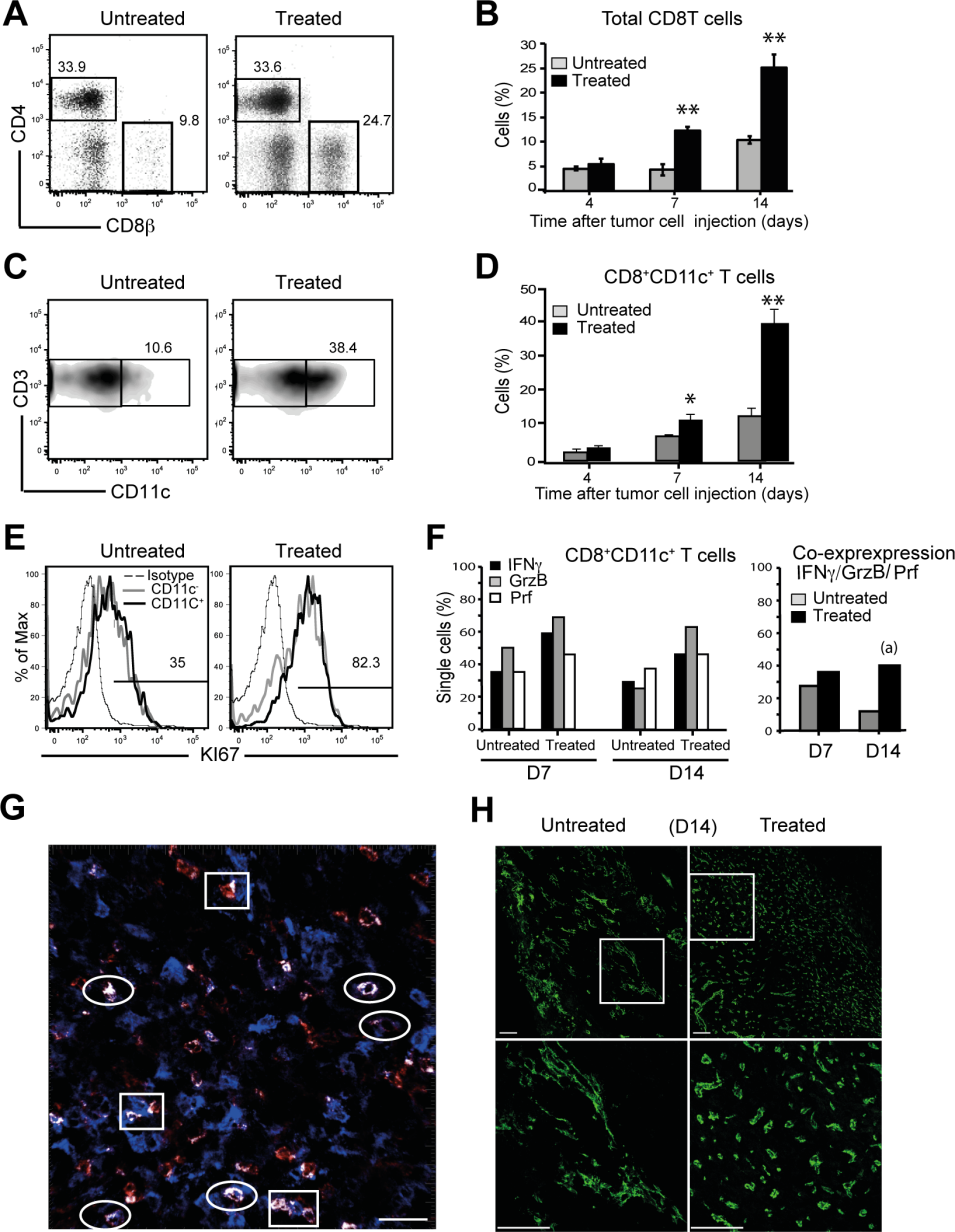
Treg depletion results in increased infiltration of CD8+CD11c+ effector T cells. (A) Representative dot plots show tumour infiltrating lymphocytes analysed by flow cytometry for cell surface CD4 and CD8β expression gated on CD3 T cells in untreated and treated tumour infiltrates (day 14). (B) Percentages of tumour infiltrating CD8 T cells in untreated (n = 4) and treated (n = 4) mice at different time points after tumour inoculation. (C) Representative dot plots of cell surface expression of CD8^+^CD11c^+^ gated on CD8 T cells. (D) Histogram shows frequencies of CD8^+^CD11c^+^ T cells in indicated groups of mice (n = 3). (E) Flow cytometry analysing proliferating cells (KI67^+^) in CD8^+^CD11c^+^ and CD8^+^CD11c^-^ T cells in the indicated groups of mice. A representative experiment of at least two independent experiments is shown, n = 3 mice per group. (F,left) Graph shows percentages of CD8^+^CD11c^+^ T cells expressing each gene and sorted from both group of untreated (119 cells) and PC61-treated (107 cells). (F,right) Graph shows percentages of CD8^+^CD11c^+^ T cells co-expressing the four genes (right). Data are obtained from two different cell-sorting (G) Frozen tumor sections obtained 14 days after tumor injection from treated and untreated mice were examined by confocal microscopy after staining with CD8β (red), CD11c (green) specific antibodies and Dapi (gray, left panels). Direct photography acquired with a 63x objective show co-staining of CD8 and CD11c antibodies on CD8 T cells. Cell surface co-expression of CD8β and CD11c (circles) and contact between CD8 T cells and dendritic cells (rectangle) are shown in white color. Data are representative of at least two independent experiments. (H) The tumour vasculature was examined by confocal microscopy following staining with CD31 specific antibody. Representative tumour sections from indicated group are shown by (A) 20x magnification; scale bar: 100 μm and (B) 63x magnification; scale bar: 100 μm. These data are representative of three independent experiments. *,p<0.05 **,p<0.001. P values were calculated using Student’s t test and (a) by Fisher’s exact test p<0.05.

Similarly to the DLN, the frequency of total CD8 T cells infiltrating the tumours was much higher in treated than untreated mice (Fig 4A and 4B). Among these, the compartment of CD8^+^CD11c^+^ T cells increased progressively between days 7–14 after tumour inoculation (Fig 4C and 4D). Moreover, CD8^+^CD11c^+^ T cells displayed functional characteristics. Based on the expression of KI67, both CD8^+^CD11c^+^ and CD8^+^CD11c^-^ T cell subsets proliferated twice as much in treated mice compared with untreated mice (Fig 4E), indicating that Treg depletion prevents all tumour infiltrating CD8 T cells from exhaustion. Next we examined the cytotoxic function using a method of single cell RT/PCR multiplex already described [18]. Single-cell RT-PCR is an appropriate tool to resolve « co-expression of the three genes associated with the cytotoxic function » (*Grzb*, *IFN*γ, *Prf*), and to assess the frequency of the cytotoxic T cells in DLNs and at the tumour sites. We observed that the proportion of single cells expressing the cytotoxic associated genes *IFNγ*, granzyme B (*Grzb*), and perforin (*Prf*) genes was similar in both groups at day 7 but however increased in *Grzb* at day 14 in treated mice. In contrast, co-expression of the three genes was markedly augmented at Day 14 from regressive tumours (Fig 4F).

Using confocal microscopy, we studied the localization of CD8^+^CD11c^+^, CD8^+^ CD11c^-^ T cells and DCs at the tumour site in both untreated and treated mice, 14 days after tumour injection. We observed a marked increase in CD8^+^CD11c^+^ T cells in the tumours of PC61-treated mice, confirming our flow cytometry data. Our images of whole tumour sections showed that the overall localization of DCs and CD8 T cells resembled the highly organized LN anatomy. In treated mice, CD8^+^CD11c^-^ cells accumulated as multiple clusters that were surrounded by DCs, while very few clusters were found in tumours from untreated mice (data not shown). Importantly CD8^+^CD11c^+^T cells (circle) co-localized with dendritic cells (Fig 4G, circles), predominantly accumulated in close contact with the DCs (rectangle) suggesting their active interaction at the tumour site. Taken together, our data provides evidence that successful control of tumour growth following PC61-treatment is the result of a strong infiltration of the tumour by poly-functional CD8^+^CD11c^+^ cytotoxic T cells.

To determine if enhanced tumour infiltration is linked to increased angiogenesis, we analysed tumour vessels by confocal microscopy using anti-CD31 antibodies in tumours from untreated and treated mice (Fig 4H). In non-treated mice, we observed the presence of large and flattened vessels. Conversely, in PC61-treated mice tumours had only few large vessels and much increased numbers of vessels with a small diameter and cuboidal morphology, consistent with the morphology of HEV. To quantitate differences in tumour vasculatures, absolute numbers and size of vessels from treated and untreated tumours were further counted on whole tumour sections. By day 14, regressing tumours showed a twofold increase of vessels per area of tumours (820 versus 391). This data demonstrates a major modification of the angiogenesis in regressive tumours, suggesting that the development of HEV favours infiltration of the tumour mass by CD8^+^CD11c^+^ effector T cells.

### DCs from Treg depleted mice induced CD8 T cell proliferation and low level of PD-1 expression

**Fig 5:**
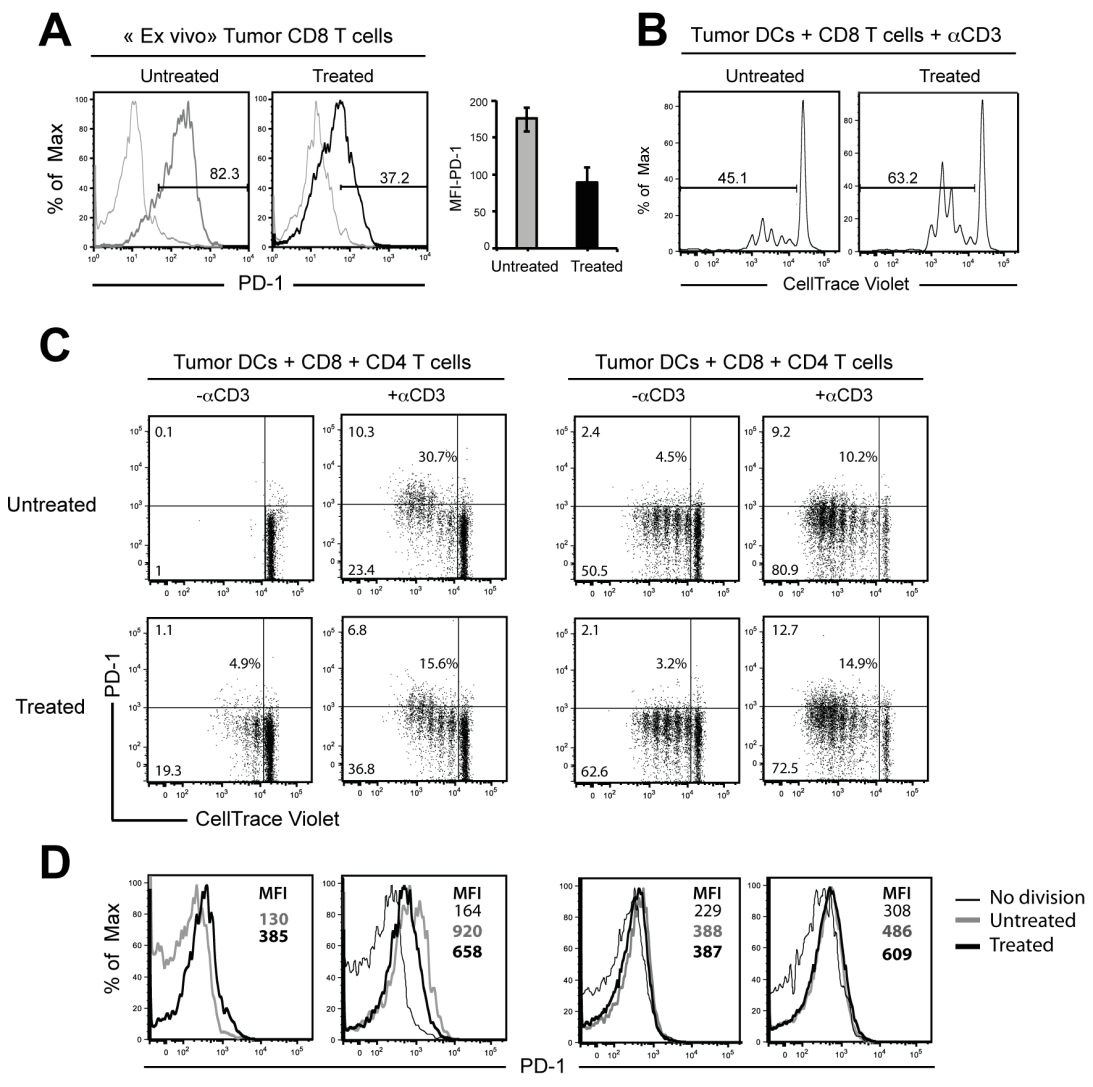
DCs from PC61-depleted mice and CD4 T cells reduce the percentages of CD8 T cells expressing high level of PD-1. (A) Histograms show cell surface expression of PD-1 on infiltrating CD8 T cells from treated and untreated tumours resected at day 14 (Left) and mean Fluorescence Intensity (MFI) of PD-1 in each group (Right). (B) CD11c^hi^MHC II^hi^ tumour DCs were sorted from treated and untreated mice and cultured 3 days with naïve CD8 T cells labelled with CellTrace violet and 0.1μg/ml of anti-CD3 mAb. (C,D) Flow cytometry analysing the expression of PD-1 on dividing cells. CD11c^hi^MHC II^hi^ DCs from Tumours (C) and CD11c^hi^ DCs from DLNs (D) were purified from untreated and PC61-treated mice and co-cultured 3 days with CellTrace violet-labelled CD8 T cells and in the presence or not with anti-CD3 mAb or CD4 T cells. Percentages of cells expressing PD-1 are represented in the middle of the graphs. (E,F) Histograms represent the level of PD-1 expression gated on all dividing cells according to indicated cultures. Results represent two independent experiments.

Published data identified PD-1 as one of the most overexpressed negative receptors by exhausted CD8 T cells in chronic infections [24] as well as anti-tumour responses [25]. Although functional effector T cells are generated during the early stage of these T cell responses, they gradually lose function during the course of the disease. Because DCs represented the obvious candidates for mediating functional effector cells, we speculate that the increased numbers of DCs observed after Treg depletion could have a positive effect on the generation of non-exhausted anti-tumour effector cells. We therefore studied PD1 expression on tumour infiltrating CD8 T cells from untreated and PC61-treated mice injected with 4T1 tumour cells. In agreement with data reported by others PD-1 was highly expressed on CD8 T cells from untreated mice (Fig 5A). In contrast, we found a marked reduction of PD-1 expression on intra-tumour CD8 T cells from treated mice. To evaluate if these differences in PD1 expression correlated with different tumour DC functional properties we first compared CD8 T cell proliferation of naïve CD8 T cells when cultured in the presence of DCs isolated from untreated and PC61-treated mice. 7 days after tumour inoculation, DCs (CD11c^hi^MHC^hi^) from treated and untreated tumours were sorted and cultured with normal CD8 T cells sorted from LNs, labeled with CellTrace Violet (CTV), division being evaluated by CTV dilutions (Fig 5B). After addition of anti-CD3 mAb, DCs from treated tumours were more efficient at stimulating the proliferation as compared with DCs from untreated tumours.

To look for a possible connection between the effect of DC stimulation and the regulation of PD-1 expression, we next analysed PD1 expression on dividing cells elicited by the different DCs. Without addition of anti-CD3 mAbs in the culture, tumour DCs from untreated mice were unable to induce CD8 T cell divisions (Fig 5C). In contrast, CD8 T cells undergo divisions in the presence of tumour DCs from treated mice, these divisions being potentiated by addition of anti-CD3 mAbs and even more by addition of CD4 T cells in the cultures. Moreover, DCs from untreated tumours led to a 2-fold increased frequency of cells expressing PD-1 upon divided CD8 T cells. In addition, as assessed by staining profile and mean fluorescence intensity (MFI), CD8 T cells stimulated by DCs from treated mice induced lower level of PD-1 expression upon dividing cells than those cultured with DCs from untreated mice (Fig 5D). In addition, comparable level of PD-1 expression were observed upon dividing cells cultured with DCs and CD4 T cells. Similarly to tumour DCs, DLN DCs isolated from treated mice and but not untreated mice induced the same pattern of low PD-1 expression upon dividing cells (Fig 5E and 5F). Importantly, CD8 T cells expressing high levels of PD1 are only generated in the absence of CD4 help showing DCs from untreated mice are not enough efficient to sustain cell division and need the CD4 help.

These results indicate that Treg depletion modifies the properties of DCs and showed that in the absence of Tregs, DLN and tumour DCs provided a stronger signal at the priming phase of activation improving the quality of CD8 T cell divisions and reducing the level of PD1.

## Discussion

The failure of immune responses against tumours has been attributed to an active suppression by CD4^+^FoxP3^+^CD25^+^ natural Tregs [26]. We have previously shown that prophylactic depletion of Tregs triggers the regression of 4T1 tumours by increasing the percentages and numbers of CD8 T cells at the tumour site [18]. Nevertheless, how *in vivo* Tregs depletion affects tumour growth and induces a vigorous effector response from the diverse repertoire of normal T cells remains poorly understood. In this study, we determined that Treg depletion by PC61 mAbs leads to tumour regression by triggering three major events: modifications of different dendritic cell subsets in the tumour draining lymph node, an enhancement of tumour neo-angiogenesis and the generation of polyfunctional cytotoxic T cells uniquely capable of infiltrating the tumour and controlling tumour growth.

PC61-Treg depletion does not fully eliminate Tregs, as CD25^-^ and CD25^lo^ FoxP3^+^ Tregs are not affected. Moreover, in the presence of the tumour, CD25^+^FoxP3^+^ Treg cells accumulate in the DLN of treated mice, reaching the same numbers as those found in untreated mice by day 14 after tumour inoculation. As this late accumulation does not prevent tumour rejection, our data indicate that CD25^+^FoxP3^+^ Treg cells only influence the priming phase of the immune response. It is well known that IL-2 is a central mediator of Treg suppression, essential for their survival and competition for limited amount of IL-2 during immune response [16, 27]. Therefore, a 2 to 3-fold reduction in the number of Tregs at the early phase of the anti-tumour immune response could increase IL-2 availability and the pool size of effector cells. Thus, the late accumulation of Treg cells would be unable to influence the late-stage immune responses and may not mediate suppression.

A previous study proposed that Tregs control the DCs homeostasis [14] as Treg ablation in Foxp3^*DTR*^ mice promotes the differentiation of high numbers of pre-DCs and cDCs from bone marrow precursors and their accumulation in LNs [13, 14]. In agreement with these data, we found an increase of resident DC subsets in DLN of PC61-treated mice as compared with untreated mice. In addition to these previous studies where increased DC subsets were not identified, we found that individual DC subsets did not behaved similarly after Treg depletion. The sequential increase of the resident DC subsets in DLN raises the question of a potential different role of each DC subset in tumour rejection. Previous data reported that DC subsets are specialized in different functions. Migratory DCs were reported to have a superior ability to generate Tregs *in vivo* [23]. It has also reported that CD11b^+^ DCs preferentially presented antigen to CD4 T cells rather than CD8^+^DCs [28]. In addition, CD11b^+^ DCs have been described as capable of inducing a protective anti-tumour immune response [29]. Lastly, LN-resident CD8^+^ DCs were associated with antigen cross-presentation and CTL priming [30]. Our data showing sequential recruitment/expansion waves of different DC subsets correlates with the sequential events occurring in the anti-tumour immune response, which lead to tumour rejection. Hence, it is likely that anti-tumour responses in treated mice are linked to the accumulated CD11b^+^ DCs that could activate CD4 T cells to produce IL-2, necessary for the efficient generation of CD8 effector T cells. Then, CD8 T cell activation would be further sustained by CD8^+^ DCs, which are known to cross-present antigens to CD8 T cells. Thus, Treg depletion promotes DC modifications as a part of a program that optimizes the microenvironment for the generation of effector T cells.

Released from Treg control, the increase of DC numbers were expected to impact the number of CD8 T cells and the generation of effector T cells. In agreement with this hypothesis, DLN increased 4-5-fold in treated mice and the number of CD8 T cells increased proportionally. This increased DLN size correlated with the DCs accumulation, suggesting that high numbers of DCs promote the recruitment of higher numbers of CD8 T cells. This recruitment of CD8 T lymphocytes should increase the probability of DC and antigen-specific T cell contact, and therefore promote the generation of higher numbers of anti-tumour-specific effector cells. This observation is in agreement with previous reports, which show that mature DCs control lymphocyte entry to LN through HEVs, modifying the lymphocyte traffic and the magnitude of the effector response [31, 32]. The high frequency of DCs at the tumour site may also explain the profound modifications of angiogenesis observed by confocal microscopy. In contrast to non-treated mice, where the tumour mass is crossed by large vessels with collapsed lumens, in treated mice the tumour becomes fully vascularized, invaded at all locations by many small permeable vessels displaying a high endothelium, with a morphology resembling that of HEV, which should promote the invasion of the tumour by effector T cells. Published studies have shown that DCs and T cells could regulate lymph node vascular growth [33, 34]. Our data indicates that this mechanism, which was only described in the LN, also applies to HEV neo-genesis at the tumour site.

Besides these modifications, we also found that Treg depletion lead to the generation of a subpopulation of anti-tumour cytotoxic effector CD8 T cells that could be identified by their expression of the integrin CD11c. These cells, that accumulated in the DLN and in the tumour of PC61-treated mice were found in close contact with DCs at tumour sites, co-expressed the cytotoxic associated genes as *INF*γ, *Grzb*, *and Prf*, proliferated vigorously and were able to kill DCs extracted from both tumours. Moreover, the adoptive transfer of only 6x10^5^ of these cells was able to control tumour growth in untreated tumour-bearing mice. Overall, these results demonstrate that the CD8^+^CD11c^+^T cells present in the DLN are cytotoxic T cells involved in tumour regression. It is possible that their expression of CD11c, an integrin involved in cell adhesion, favour their migration into the tumour mass.

Using Foxp3^DTR^ mice to deplete Tregs, previous studies have observed an acute lymphopenia immediately after DTX injection and this was transient since T cell numbers recovered by day 6 [35]. Although this could explain the increase of DCs and CD8 effector T cells, it is not consistent with our data showing no change in DCs and T cell numbers 5 days after PC61 treatment of normal mice (data not shown). There are differences in our model and theses studies. PC61 treatment is not fully effective because not all Treg cells are depleted. Moreover, PC61 treatment seems to be rather long lasting compared with the transient depletion using DTx-based mouse model [36, 37]. Therefore we can only speculate that this increase of DCs in the context of tumour inoculation is linked to inflammatory microenvironment. However, we cannot fully exclude an impact of an undetectable mild lymphopenia and probably both mechanisms are implicated. Importantly, our work provides evidence that Treg depletion induces the generation of non-exhausted anti-tumour effector T cells. Indeed, at tumour sites, total CD8 T cells express low levels of PD1 in Treg depleted mice, while PD1 is highly expressed on effector cells from untreated mice. PD-1 is a critical negative co-receptor that is highly expressed on chronically stimulated CD4 and CD8 T cells and is involved in peripheral tolerance [5]. Tumour-infiltrating T cells in humans and mice were reported to be “exhausted”, i.e., functionally impaired due to their high expression of PD-1 [4]. Furthermore, PD-1 expression on tumour-infiltrating CD8 T cells in humans was shown to identify clonally expanded tumour-specific cells [25]. Consistent with these data, we found that PD1 was highly expressed on cells of untreated mice that weakly proliferated Thus, our *in vivo* data establishes a direct link between Treg-mediated suppression and the expression of high levels of PD-1 on exhausted cells.

The finding that in PC61-treated mice, all CD8 effector cells accumulate inside the tumour with low expression of PD-1 raises the question of how these cells are not chronically stimulated. It is tempting to speculate that chronic stimulation does not occur because PD1^lo^ cells have the capacity to kill DCs presenting the tumour antigens. This effect would regulate the immune response by reducing antigen presentation to expanded CD8 T cells and prevent their chronic stimulation and exhaustion. This is supported by previous reports in which the killing of DCs was shown to be an important mechanism for the regulation of T cell responses [38–40] and our own data showing CD8^+^CD11c^+^ T cell degranulation upon interaction with tumour DCs. Our results also highlight a correlation between Treg levels, CD4 help/IL-2 availability, DC properties, and the expression of PD1. It was previously reported that PD1 expression inhibited the IL-2 autonomous production by CD8 T cells [41], yet a possible effect of modified DCs on PD-1 expression was not addressed. Our findings define a role of DC modifications in modulating PD-1 expression during anti-tumour responses in Treg-depleted mice. Thus, our data underline the effect of Treg depletion on DCs and their capacity to efficiently activate CD8 T cells and regulate their PD1 expression during the anti-tumour immune response.

By combining the multiple reports cited above and our novel data, we propose a model of tumour regression providing a possible explanation for the complex events following the transient reduction of Tregs by PC61 treatment in tumour-bearing mice. The modifications of DC populations would be responsible for most of the modifications of the anti-tumour response leading to tumour rejection. Thus, the recruitment of CD11b^+^DC subset as well as the reduced Treg numbers would decrease considerably the Treg/DC ratio, providing up-regulation of co-stimulatory molecules, followed by increased CD8 T cell recruitment in DLN. These events should favour the efficient activation and expansion of CD8^+^CD11c^+^ cytotoxic T cells, activation of which in the presence of IL2 induces their rapid expansion and avoids PD-1 expression. A higher capacity of these cells to divide and invade the tumour, together with an extensive angiogenesis, would allow antigen-specific cell expansion and elimination of the tumour mass. Importantly, these CTLs could prevent chronic T cell stimulation, high level of PD1 expression and functional inactivation by reducing antigen loads through killing tumour cells and the DCs expressing tumour antigens.

Our data highlight the effect of Treg depletion on tumour regression and the major role of three DC subsets that act at different time points of the anti-tumour response. Therefore, our results delineate an additional level at which DCs induce complete tumour regression by orchestrating all events leading to fully differentiated CD8^+^CD11c^+^PD1^lo^ effector cells and their migration into tumours. With regard to cancer, new approaches for cancer therapies will be facilitated by the identification of cellular pathways involved in the role of Treg cells in the evasion of tumour immunity. Thus, the sequential and differential impact of DC subsets may guide the design of new protocols for partial Treg depletion and DC generation, in order to develop more efficient anti-tumour therapy.
