# Correction: Depletion of Regulatory T Cells Induces High Numbers of Dendritic Cells and Unmasks a Subset of Anti-Tumour CD8^+^CD11c^+^ PD-1^lo^ Effector T Cells

**DOI:** 10.1371/journal.pone.0171373

**Published:** 2017-01-27

**Authors:** Nicolas Goudin, Pascal Chappert, Jérome Mégret, David-Alexandre Gross, Benedita Rocha, Orly Azogui

Fig 5 appears incorrectly in the published article. Please see the correct Fig 5 and its caption here.

**Fig 5:**
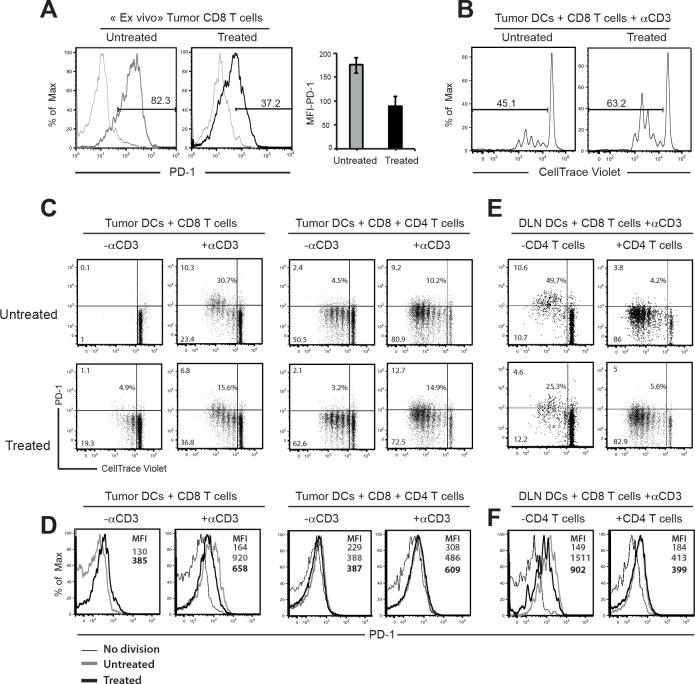
DCs from PC61-depleted mice and CD4 T cells reduce the percentages of CD8 T cells expressing high level of PD-1. (A) Histograms show cell surface expression of PD-1 on infiltrating CD8 T cells from treated and untreated tumours resected at day 14 (Left) and mean Fluorescence Intensity (MFI) of PD-1 in each group (Right). (B) CD11c^hi^MHC II^hi^ tumour DCs were sorted from treated and untreated mice and cultured 3 days with naïve CD8 T cells labelled with CellTrace violet and 0.1μg/ml of anti-CD3 mAb. (C,D) Flow cytometry analysing the expression of PD-1 on dividing cells. CD11c^hi^MHC II^hi^ DCs from Tumours (C) and CD11c^hi^ DCs from DLNs (D) were purified from untreated and PC61-treated mice and co-cultured 3 days with CellTrace violet-labelled CD8 T cells and in the presence or not with anti-CD3 mAb or CD4 T cells. Percentages of cells expressing PD-1 are represented in the middle of the graphs. (E,F) Histograms represent the level of PD-1 expression gated on all dividing cells according to indicated cultures. Results represent two independent experiments.
